# Digitalisation promotes adoption of soft information in SME credit evaluation: the case of Indian banks

**DOI:** 10.1007/s42521-023-00078-w

**Published:** 2023-04-21

**Authors:** Nimbark Hardik

**Affiliations:** Université Côte D’azur, Institut d’Administration des Entreprises, Campus Saint-Jean d’Angély, 5 avenue du 22e, B.C.A, 06300 Nice, France

**Keywords:** Big data, Credit evaluation process, Digitalisation, Organisational adoption, SME financing, Soft information, D22, D82, G21, G32, O33

## Abstract

Small and Medium Enterprises (SMEs) account for half of the employment in developing economies and are a significant part of their economic growth. In spite of this, SMEs are under-financed by banks, which have been disrupted by financial technology (fintech) firms. This qualitative multi-case study examines how Indian banks are utilising digitalisation, soft information, and Big data to improve SME financing. The participants shared their insights on the way banks adopt digital tools, sources of soft information (e.g., customer and supplier relationships, business plans), and factors that influence the implementation of Big data in the SME credit evaluation process. The major themes include: banks are improving SME financing operations through digitalisation, and IT tools can verify SME soft information. Soft information attributes that emerge from addressing SME information opacity include supplier relationships, customer relationships, business plans, and managerial successions. For SME credit managers, developing partnerships to access publicly available soft information created by industry associations and "online B2B trade platforms" is a high-priority recommendation. To enhance the efficiency of SME financing, banks should obtain the consent of SMEs before they access their private hard information through trade platforms.

## Introduction

### SME’s information asymmetry: the market for lemons

This study is based on the theory of information asymmetry in markets developed by George A. Akerlof (1970). According to Akerlof (1970), credit markets in developing countries often exhibit strong indicators of the Lemons Principle (Akerlof, 1970). It is a challenging task for bankers to identify good SMEs for financing, which is equivalent to identifying peaches from lemons. According to Froot and Stein (1998), "information is a crucial input for the banking business, especially risk management, capital budgeting, and capital structure policies" (Godbillon-Camus & Godlewski, 2013, p. 3). Diamond (1984) adds that the primary function of financial intermediaries in financial markets is to overcome borrower information asymmetry (Godbillon-Camus & Godlewski, 2013).

Several operational issues exist when banks finance SMEs: Credit risk, high service costs, and lower revenue per account (Owens et al., 2017). Bankers use quantitative (hard) and qualitative (soft) information to assess credit risk. According to Liberti and Petersen (2018), soft information can be defined as "Information that cannot be summarised completely in a numeric score, requires context, and becomes less useful when removed from the context" (Liberti & Petersen, 2018, p. 2). According to ICCR (2018), lack of credit data is one of the major obstacles to financing individuals and SMEs in developing countries (La Torre et al., 2010; Owens et al., 2017). A number of industries, including the financial services industry, have been disrupted by digital technology, driven by advances in mobile computing. Digital data have been expected to double every 2 years (Owens et al., 2017), and digitalisation is causing banks to rethink the core products and value chains of their business (Urs Gasser et al., 2017).

### Mitigation of SME's information opaqueness through digital data

Despite employing a large percentage of the labour force in developing countries, SMEs receive limited external funding compared to large firms, which leaves them with a financing gap. As indicated in the ICCR’s (2018) report, formal SMEs account for 33% of GDP and 45% of employment, whereas these statistics are significantly higher if MSMEs and the informal sector are included. According to the World Bank Group, approximately half of the estimated 400 million small- and medium-sized enterprises (SMEs) in developing markets have unmet credit needs totalling USD 2.1 to USD 2.6 trillion annually (Owens et al., 2017).

**Fig. 1:**
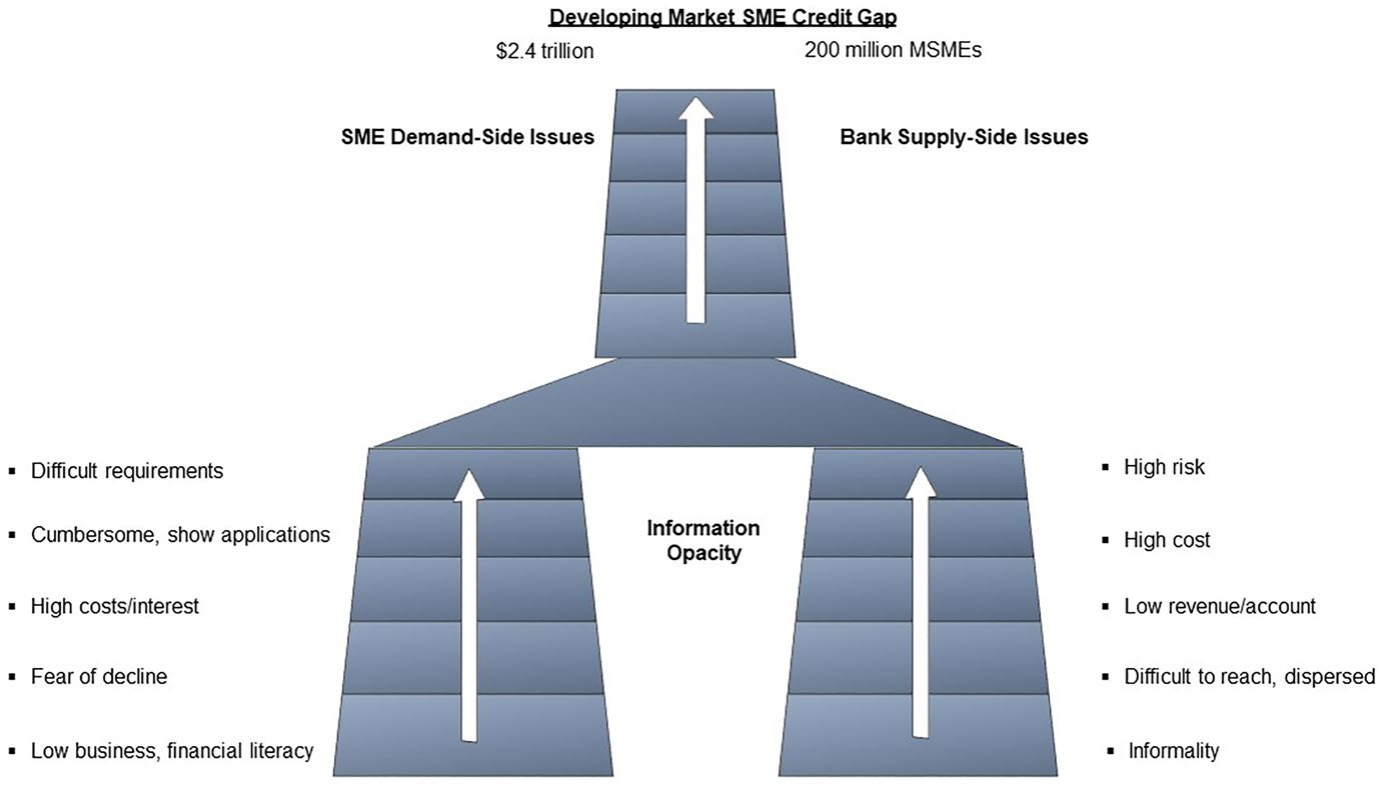
SME financing market—supply side vs demand side (Owens et al., 2017, p. 16)

Regulatory, academic, and market views improve knowledge of disruption phenomena. In banking and finance, innovation is driven by data (Walliser & Mignon, 2015). In the context of digitalisation and Big data adoption by Indian banks, this study explores three questions about how new players and incumbents can address the pressing challenges of SME financing (Fig. 1). How is digitalisation helping banks in addressing the operational challenges of SME credit evaluation? How does soft information address SMEs' information opaqueness? How is the Big data helping banks in assessing credit risk?

### Research gap

The initial literature review suggests that disruption in the financial services industry has unprecedented consequences. These predictions, however, have not been shown to manifest themselves in the banking industry or affect specific business areas, including financing for small- and medium-sized businesses. I develop a broad understanding of the disruptive phenomenon in the financial services’ industry by examining regulatory publications, academic articles, and practitioner perspectives. Through this process, four research gaps are identified.

#### SME’s credit evaluation and addressing constraints of soft information

This research gap examines the constraints of soft information in the context of SME credit evaluation. The banks rely on quantitative and qualitative information to evaluate SME credit. It has been extensively discussed in the literature that soft information is important in SME financing, and its limitations are that it is unverifiable, unquantifiable, and sometimes subjective (Berger & Udell, 2006; Yosano & Nakaoka, 2019). There is, however, little evidence of how banks overcome these limitations and whether they use the hardening of soft information (Uchida et al., 2008).

#### SME credit evaluation and content of soft information

A second research gap examines the content of SMEs' soft information, also known as hard and soft information in the context of SME credit evaluation. To address SME information opaqueness, soft information has been widely discussed. Furthermore, it is evident that soft information enabled lenders (i.e., banks) to identify potential SMEs from the market by applying a risk-based approach. There is, however, a lack of research on the content of soft information (Del Gaudio et al., 2020), i.e., which specific SME soft information attributes are most important for lenders.

#### SME credit evaluation and adoption of digital technologies

The third research gap examines banks' perceptions of digitalisation and the adoption of digital technologies in the context of business process digitisation. There is limited evidence of lenders' or banks' perceptions about the use of digitalisation and Big data in SME credit evaluation activities (Baig et al., 2019; Uchida et al., 2008).

#### Digitalisation phenomenon impacting banks

Last but not least, limited academic research is carried out about the impact of digital technologies on banks. Fintech firms disrupting payment products have eroded about 10% of banks' profit margins, according to a recent study. However, there is limited evidence on how banks or incumbents could be responding to digitalisation and the adoption of digital technologies. I have decade-plus experience in the banking industry that contributes to confirming the gap in the literature. The conceptual framework and research questions are refined as a result of the identified research gaps and limitations. This study examines the credit evaluation process for banks that primarily financed SMEs. Various factors related to credit evaluation are examined in this study, including information technology, financial innovation, Big data, and soft information.

### Research objectives

According to the study by Kumar and Rao (2015, 2016), almost 92% of SMEs in India have no access to traditional sources of finance; they are reliant on informal sources of funding or self-financing (Kumar & Rao, 2015, 2016). As primary lenders in the market, banks face productivity challenges while addressing SMEs' information opaqueness using soft information. EBA (2018) stresses that digitalisation and the adoption of large amounts of data are on the rise among financial institutions, as evidenced by Petersen and Rajan (2002) that IT adoption improves productivity (EBA, 2018; Petersen & Rajan, 2002). Moreover, Beck et al. (2016) state that financial innovation has the potential to improve productivity for banks, particularly in developing countries with stricter regulatory guidelines (Beck et al., 2016).

As IT advances, Petersen and Rajan (2002) predict that lenders would ignore small and opaque companies due to the difficulty of incorporating soft information into computer-based credit decisions. However, Filomeni et al. (2016) suggest the use of information technology to harden soft information within a large European bank, extending soft information boundaries in the process. Likewise, Cornée (2019) emphasises that soft information collection generates substantial labour costs. However, with new IT developments that increase soft information's economic value, soft information collection costs decrease. The development of hard and quantifiable information by IT in banks has positively impacted business processes. Meanwhile, IT adoption allows banks to process and transmit quantitative information for decision-making purposes. It is clear that IT adoption should be carefully incorporated into this study in light of the importance of IT in the lending process activities, i.e., production of soft information, transmission of soft information, and hardening of soft information.

Therefore, the aim of this qualitative multi-case study is to gain an understanding of Indian bankers' perceptions about the adoption of digital technology, soft information, and Big data technology in the process of evaluating SME credit. In the study, empirical evidence about a bank's perspective on SME financing is sought. The research gaps are addressed by articulating three research objectives. The first objective is to assess the level of IT adoption among large and small banks in India for evaluating SME credit scores. The second one is to understand the usage and content of SME's soft information that facilitates addressing SME's information opacity. The third objective is to identify the perceived internal and external factors that influence the adoption of Big data by banks.

The research objectives are translated into research questions

- Research question one (RQ1), how is digitalisation helping banks in addressing operational challenges of the credit evaluation process in financing SMEs?
- Research question two (RQ2), how does soft information address SME information opaqueness to identify potential SMEs?
- Research question three (RQ3), how are banks benefiting from the implementation of Big data in creditworthiness assessment?

### Research scope, results, and contributions

The banking and finance industry is highly regulated as a systemically important industry. There are strict confidentiality and non-disclosure rules that govern the industry. Therefore, researchers are unable to access the most current data and information from bankers and lending organisations. Another important aspect is to look for financial innovation in developing country banks whose SME financing gap is very large. Hence, the study aimed to combine data from banks regarding their SME credit evaluation processes with views from regulatory and policy development bodies regarding digitalisation, soft information, and the use of Big data. The approach contributed to the development of a comprehensive understanding of the market based on empirical evidence.

This study identified seven themes that are comparable with the literature and are in accordance with the conceptual theoretical framework of this study. The most significant themes are: banks are enhancing the productivity of SME financing operations through digitalisation, and SME soft information can be verified using IT tools. A discussion of the benefits and influences of SMEs' soft information followed, in which the bankers stressed the importance of the "weighting of soft information" and the "true picture of creditworthiness" for the evaluation of SMEs' creditworthiness.

The study results reveal significant evidence of banks "hardening soft information" during the generation of soft information. It is also evident from the results of the study that large banks are more likely than small banks to adopt information technology, and that bigger firms (regardless of their market power) are more likely to innovate in terms of SME financing (Akhavein et al., 2005; Marinč, 2013; Schumpeter, 1983).

This study provides an important contribution to theoretical knowledge by proposing a conceptual theoretical framework. Using the framework, researchers are able to evaluate contemporary and critical research areas, such as digitalization, credit evaluation, soft information, and Big data technologies within financial institutions. In addition, a significant contribution to theoretical knowledge is provided by commonly perceived soft information factors among Indian bankers, which are "networks or alliances/partnerships" as well as "business and management leadership". Bankers can distinguish good SMEs from bad ones with the help of these factors and reduce lender losses as a result.

Furthermore, the study contributes to the emergence of a common perception of risks in adoption of Big data tools among diverse yet well-connected groups, especially SME bankers and regulatory specialists. Data privacy, data security, and transparency are among the most commonly perceived risks when banks adopted Big data tools to evaluate SME credit.

## Theoretical foundation

In recent years, IT advancements that emphasise hard and quantifiable information have positively impacted a significant part of the banking industry's business process (Marinč, 2013). Also, the adoption of IT helps banks efficiently process and transmit data that is useful for decision-making. IT facilitates better communication and information availability, thus improving banking activities (Marinč, 2013). In addition to improving the quality and quantity of information available to banks, IT has also facilitated the creation of Big data for banks. The adoption of retail credit scoring models has also been accelerated by the use of information technology, while SME financing—a very IT-intensive process—has become increasingly digitalised as well. Therefore, the study examines the "SME credit evaluation process at banks" and narrows the analysis to a specific business process.

**Fig. 2:**
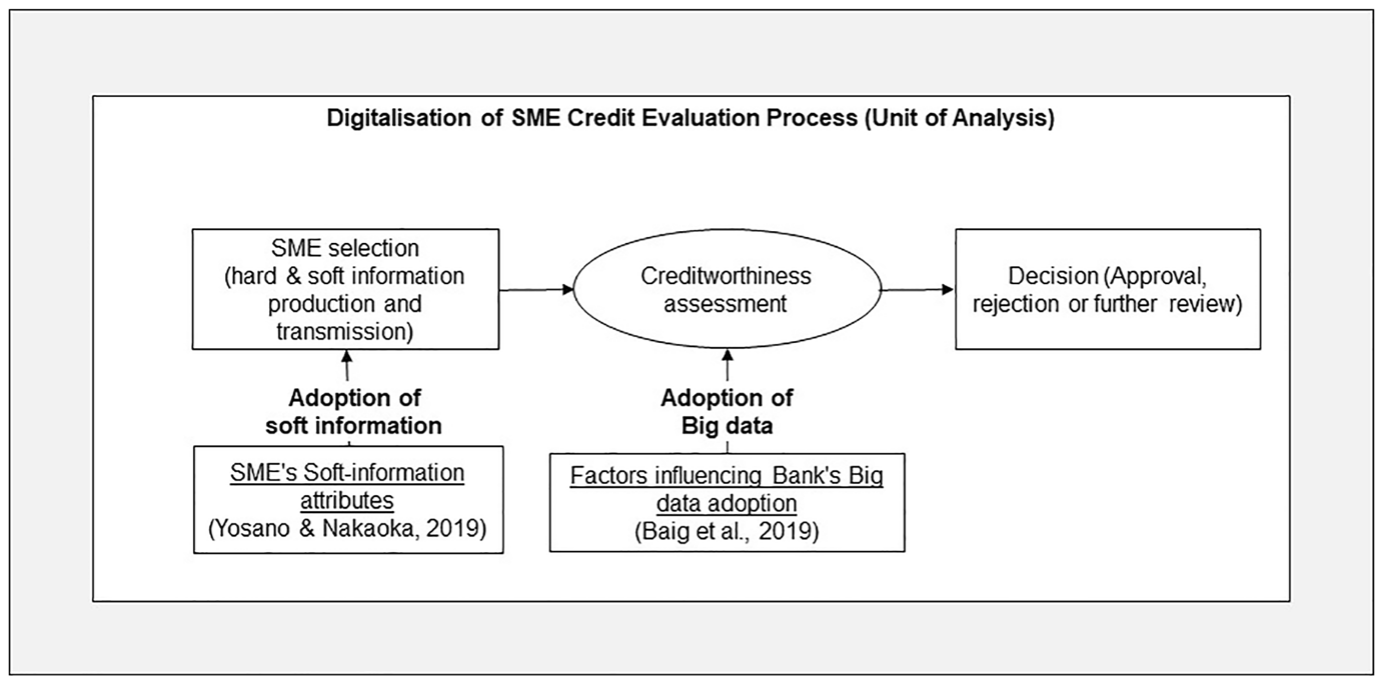
Conceptual theoretical framework of this study based on organisational adoption (Rogers, 2005; Yosano & Nakaoka, 2019; Baig et al., 2019) of advanced IT. Source: By the author

Among academics, financial regulators, and policymakers, the concept of credit scoring has long been well established. As a result of digitalisation, digital data development, and Big data technologies, the role of information technology and data would need to be analysed. It is, therefore, necessary to develop a specific framework or theory that could support the discussion of a variety of diverse yet interconnected topics within the context of "SME credit evaluation at banks". An SME credit evaluation process consists of three steps: the SME's credit application (including the production of hard and soft data), the credit evaluation and score generation, and the decision (approval, rejection, or further review) (Fig. 2).

To develop the conceptual theoretical framework, Rogers (2005)'s "theory of organisational adoption/implementation" is used as a theoretical foundation. The proposed conceptual framework includes Yosano and Nakaoka's (2019) framework when considering soft information to assess the adoption of digital technologies within an organisation and its business processes. During the credit evaluation process, it assists in evaluating the production, transmission, and content of soft information by small- and medium-sized businesses. To determine the factors influencing the adoption of Big data technology in credit evaluation, Baig et al.’s (2019) framework is incorporated into the conceptual framework.

### Innovation adoption in an organisation

According to (Rogers, 1995), adoption in an organisational context refers to an organisation's degree of awareness of or commitment to a particular technology or idea. However, during the diffusion phase, the technology is sparing the population, individuals, groups, and organisations (Rogers, 1995). The book by Professor Zaltman et al., (1973) has been regarded as a milestone in the field of organisational innovation. They suggest examining organisational adoption from the following perspectives: adoption (deciding to use an innovation) or implementation (implementing an innovation) (Rogers, 2005). This study assesses the adoption of innovation within each organisation based on the findings.

**Fig. 3:**
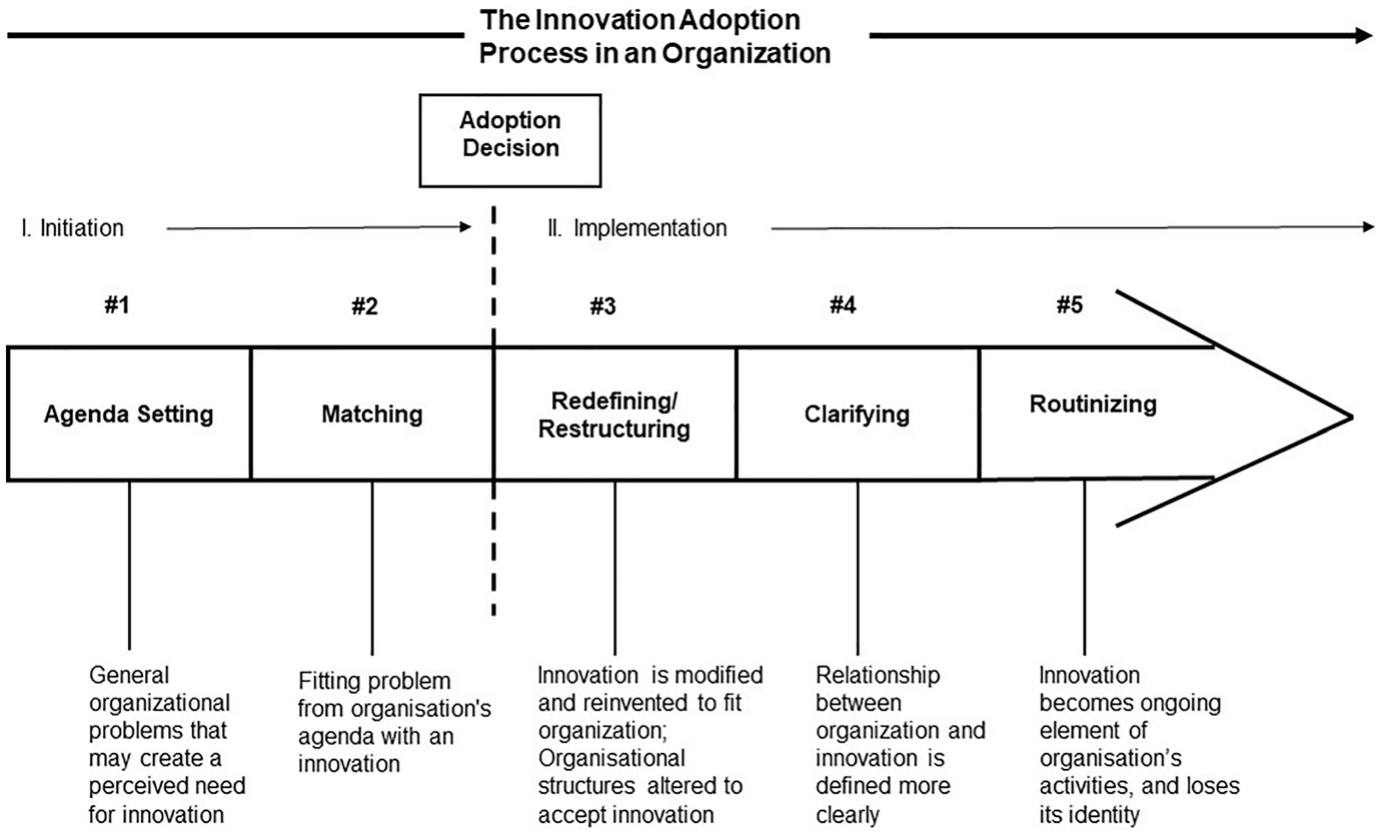
Five stages of the innovation process in an organisation (Rogers, 2005, p. 537)

In accordance with Rogers (2005), innovation has typically been divided into two broad phases, initiation and implementation. Initiating an innovation involves gathering information, conceptualising it, and planning it, leading up to its adoption. A third component of the implementation process is the activities, actions, and decisions involved in implementing an innovation (Rogers, 2005) (refer to Fig. 3).

Innovation within an organisation requires mutual adaptation to be implemented. In addition to adapting to the existing organisational structures and practices, innovation transforms them as well (Rogers, 2005). Organisational innovation is largely influenced by market-driven factors. A theoretical sampling variable is used to compare participants'/bankers' insights into the adoption of digital technology within each organisation. Participants are expected to provide insights into the adoption of digital technology within each organisation. This study identifies participants who have experience in SME lending, understood business technology within the bank, and experience different levels of digital adoption in different banks in India.

### Adoption of SME’s soft information and influence of digital technologies

According to Liberti and Petersen (2018), soft information can be defined as "Information that cannot be summarised completely in a numeric score, requires context, and becomes less useful when removed from the context" (Liberti & Petersen, 2018, p. 2). A great deal of research has been conducted on "soft information" over the last 2 decades. Researchers have highlighted many benefits or advantages of soft information, ranging from reduced interest rates to increased access to credit for borrowers to greater efficiency in integrated risk management (refer to Table 1). Further, soft information has been said to have an important role in facilitating the financing to SMEs who are perceived as opaque and possessing poor quality hard information.

**Table 1:** Summary of discussion about SME soft information characteristics and findings

Soft information characteristic	Findings/argument
Relation between soft information and SME lending	"Compared to other lending products by banks, the distinguishing characteristic of SME lending is that it heavily depends on soft information that a third party cannot verify" (Stein, 2002, p. 1892)
Soft information storage and transmission	"Soft information attributes cannot be unambiguously documented in a report by a loan officer and pass on to his superiors" (Stein, 2002, p. 1892)
Soft information collection in a bank-borrower relationship	"Soft information provides access to private and confidential information, superior to publicly available information" (Berger & Frame, 2007, p. 12)
Soft information is tightly linked to the environment and context where it is produced	"In banking, it is produced through multiple interactions over time, giving access to private and confidential information" (Godbillon-Camus & Godlewski, 2005, p. 7)
Soft information increases the predictive capacity of hard information but remains non-verifiable	"Due to non-verifiability property of soft information, it is susceptible to manipulation by loan officers" (Godbillon-Camus & Godlewski, 2005, p. 7)
Soft information takes significant time to accumulate	"Financial institutions or banks address SME’s opacity problem by relationship lending based on soft information collected through contact with the firm’s owners, managers, and other local community members over time" (Berger & Frame, 2007, p. 5)
Soft information storage and transmission	"Soft information is not easily observed, verified, or transmitted to others" (Berger & Frame, 2007, p. 5)
Soft information is mostly qualitative and subjective	"Specific knowledge of the quality of the firm’s management, its competitive position, and the daily business are mostly qualitative and subjective" (Godbillon-Camus & Godlewski, 2013, p. 5)
Soft information is mostly communicated as text	"Soft information in text form are opinions, ideas, rumours, economic projections, statements of management’s plans, and market commentary" (Godbillon-Camus & Godlewski, 2013, p. 5)

Even though empirical research has extensively examined the importance of soft information in SME financing, very few papers have attempted to explore the content and value of soft information. In Chen et al. (2015), results are the first attempt to identify SMEs' soft information factors instrumental in SME financing based on the findings of a Taiwanese finance company. The study by Chen et al. (2015) identifies three types of soft information factors relevant to SMEs: management and organisational capabilities, regulatory and macroeconomic factors related to borrowing firms' industries, and external relationships with their suppliers (Chen et al., 2015). Several studies have provided examples of soft information; however, few have identified specific soft information factors. In addition, the existing literature discusses extensively how soft information can be transmitted and used; however, it lacks sufficient evidence regarding how soft information is produced/created from the lender's perspective.

The book co-authored by Yosano and Nakaoka (2019) confirms soft information factors, their attributes, and their significance to small- and medium-sized businesses. In Japanese regional markets, Yosano and Nakaoka (2019) examines soft information utilisation for SME lenders in Japanese regional markets. Three soft information factors and 22 attributes are identified by Yosano and Nakaoka (2019) through the analysis of 54 non-financial items. These are categorised into organisational systems, networks or alliances/partnerships, and business and management leadership. Based on discussions with participants, the study identifies soft information factors and attributes that are perceived to influence SME credit evaluation.

### Adoption of Big data and influencing factors

EBA (2018) endorses the use of Big data in the credit scoring process, highlighting the key benefits such as (a) improving insights from existing data sources, (b) automating the credit decision process, and (c) exploring new data sources when it comes to credit scoring (EBA, 2018). According to ICCR (2018), the MSME customer segment would benefit the most from the use of a type of Big data called "alternative data", which can have a significant impact on developing countries (ICCR, 2018). In a similar vein, EBA (2018) points out that as Big data technologies continue to advance, a wide variety of data, including social media, public information, location data, and web-search history, can be used to extract value. A broader array of qualitative or soft data facilitates assessing a prospective borrower's behaviour, willingness to pay, and responsibility and predicting a default probability (EBA, 2018).

In this study, Big data adoption has been an important topic. EBA (2018) notes that Big data in credit scoring could provide financial institutions with a prudential opportunity. Furthermore, Hopp et al. (2018) identify "innovation from data" and "Big data in product development" as potential future research topics (Hopp et al., 2018). In banking, Big data is defined as "regularly expanding information that is difficult to process through traditional data storage" (Baig et al., 2019, p. 3). In recent years, Big data adoption has been a novel concept, and several researchers have attempted to define it. Baig et al. (2019) summarise some of the recent definitions of Big data adoption. On the basis of well-studied technology adoption frameworks, Baig et al. (2019) identify factors influencing the adoption of Big data in organisations. These factors are categorised into four categories as follows: technology (eleven factors), organisation (six factors), environment (eight factors), and innovation (seven factors) (Baig et al., 2019). This study identifies the perceived factors influencing Big data adoption in SME credit evaluation based on discussion with participants.

**Fig. 4:**
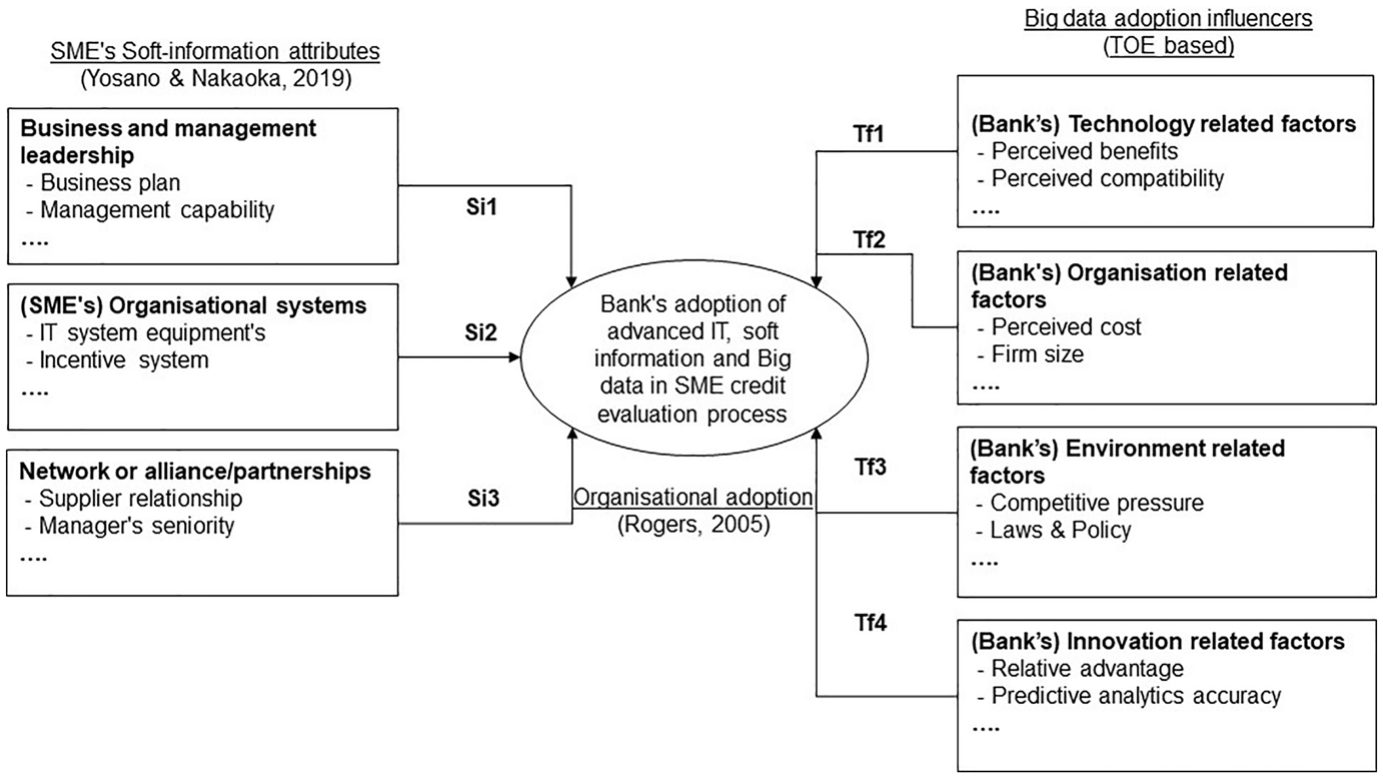
Conceptual theoretical framework of this study with a detailed view. Source: By the author

Referring to Fig. 4, this study addresses the research questions by utilising a conceptual framework which logically amalgamated one theory with two frameworks from the existing literature. Conceptual frameworks represent a summary of the literature review and the understanding built upon it. In addition, conceptual frameworks support future research and discussion of findings.

## Methodology

### Research methodology

A number of factors are considered in evaluating the appropriateness of the research method. In relation to the quantitative method, Yilmaz (2013) defines it as the use of quantitative measures for collecting and analysing data to make predictions about the results (Yilmaz, 2013). The objective of quantitative research is to test a hypothesis on the basis of numerical data using a survey (Venkatesh et al., 2016). Contrary to popular belief, the purpose of this study is not to collect quantitative data but rather to gather detailed expressions of individual participants. Consequently, the absence of pre-existing theory renders quantitative designs unsuitable. A qualitative approach using a multi-case study design is selected as the most appropriate methodology for this study, as theory is developed while perspectives are gathered from bankers who faced different challenges in lending to small- and medium-sized businesses in the organisation’s credit evaluation process (Ridder, 2017). The qualitative method enables researchers to gain a better understanding of an occurrence from the perspective of the people who experience it (Vaismoradi et al., 2015). During the data collection process, bankers and regulators are interviewed via video-recorded interviews (Brinkmann, 2014; Harrison et al., 2017). A triangulation of data is accomplished by considering regulators’ overarching perspective on the banking industry, the progress of technology, and perceived risks associated with lending to small businesses.

### Research design and scope

In this study, a sound research design is developed using the research onion map by Saunders et al. (2016). This study employs the interpretivism philosophy to understand subjective and socially constructed meanings expressed about the digitalisation phenomenon within the context of SME credit evaluation processes. Furthermore, Denzin and Lincoln (2011) state that qualitative research has predominantly been associated with interpretive philosophy (Denzin & Lincoln, 2011; Milliot & Freeman, 2015; Saunders et al., 2016). Inductive theory development is employed in this study to “develop a more comprehensive theoretical perspective that addressed literature limitations and gaps by using a naturalistic and emergent research design” (Saunders et al., 2016, p. 168). Based on open-ended questions, the purpose of the study is to gather feedback as part of its exploratory nature. Furthermore, all three research questions begin with the word “How” in an effort to understand how soft information, Big data, and digital transformation play an important role in a bank’s evaluation of SME credit (Saunders et al., 2016).

As the study employs a single method of data collection, “semi-structured interviews”, as well as a qualitative analysis procedure, it has been referred to as a monomethod study (Saunders et al., 2016). Second, “case study” is chosen as a research strategy, since “case” can be used to refer to individuals (for example, loan officers), groups (for instance, credit assessment units), organisations (for example, banks), processes (for example, credit evaluation processes), as well as many other types of case subjects (Saunders et al., 2016). Furthermore, the study has a cross-sectional time horizon, as it is performed at a particular time in history.

**Fig. 5:**
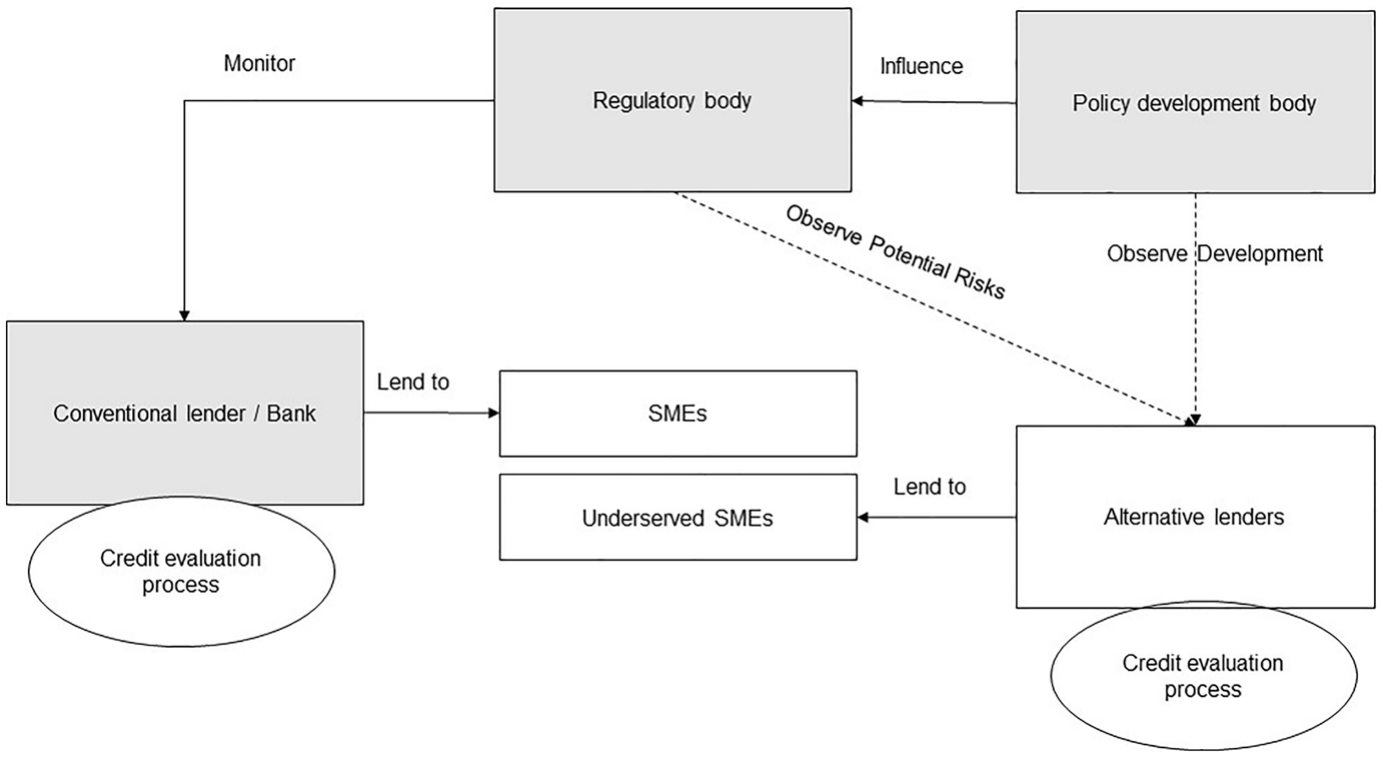
Scope of research, stakeholders involved, and their relationship. Source: By the author

Referring to Fig. 5, secondary research contributes to developing the research scope, identifying key stakeholders, and defining their relationship within the Indian SME financing industry. As indicated in the above diagram, regulators continue to monitor banks as a systemically important industry. While monitoring market development, policy development bodies influence the development of laws and regulations which directly impact banks at the same time. Symbiotic relationships exist among banks, regulators, and policymakers. The study examines conventional banks, regulatory bodies, and policy development organisations, highlighted in the grey coloured box in Fig. 5.

### Population and sample

A multi-case study design was used to provide analytic generalization (for example, extending beyond the setting of a particular case or experiment; Yin, 2011). Analytic generalization is accomplished through the use of a multi-case study, which parallels experimental research (Yin, 2011). A comparative case study (multi-case study) is selected to maximize the likelihood of generalising results to similar organisations. Therefore, participants are selected based on the size of their bank’s SME financing portfolio and current SME financing activity in the market. In India, 36 banks are publicly listed, with approximately one-third owned by the government and the remaining by the private sector. Top-10 banks in the Indian banking sector represent approximately 95% of the market capitalisation. Seven participants from the banking community agree to participate through semi-structured interviews. The banks of the participants represent approximately 65–70% of the market capitalisation. In addition, five participants from regulatory bodies agree to participate with direct exposure to overseeing SME lending activities in the country.

### Data collection, processing, and analysis

Interviews are conducted via video conferencing with participants' permission for recording and analysis. During the first half of 2021, in-person interviews are avoided due to COVID-19 protocols. Following the interview guide, the discussion lasted from forty-five minutes to an hour and fifteen minutes. During this session, the participants discuss facets of the SME credit evaluation process in a bank, including soft information factors, the influence of Big data technology, and the relationship between the research constructs. The data triangulation is addressed by arranging two data sources: Seven SME financing bankers and five regulatory specialists. In response to the initial analysis of participant feedback, follow-up questions are raised to better understand two emerging aspects of the data, namely the perceived benefits of soft information and the perception of soft information’s impact on credit conditions.

The data processing stage involved deconstruction, coding, and assembling of data using interview questions and then identifying emerging themes and relating these themes to the research question. The aim of the qualitative study is to develop relationships and patterns revealing the meaning of the responses to the instrument (Harrison et al., 2017). The purpose of the study is to analyse verbal narratives and documented responses and to develop relationships and patterns that would provide insight into the meaning of the responses given to the instrument (Denham & Onwuegbuzie, 2013; Harrison et al., 2017). Following the completion of the interviews, the analysis has begun. Transcripts are generated following the completion of the interviews using OTTER.AI, then validated and corrected against the recording. The transcripts are emailed to participants for review and corrections, as well as peer-reviewed by DBA cohorts for any inaccuracies.

Thematic, content, and narrative analysis is carried out, respectively, to uncover different levels of insights. The thematic analysis follows Strauss and Corbin’s (1998) model where the analysis is divided into three steps: open coding, axial coding, and selective coding. Open and axial coding is used to separate the content from the transcript texts and to categorise the responses to the questions (Saldaña, 2013). Open coding is an analytic process in which concepts are identified and their attributes and aspects are discovered in data. Through open coding, patterns within data could be identified and analysed, which facilitated the identification of emerging themes (Gläser & Laudel, 2013; Saldaña, 2013). Axial coding, the second phase, reassembles fragmented material in new ways by connecting a category with a subcategory. Selective coding, the third phase, incorporates and develops the theory. A theme is formed by connecting all other categories to the core category to conclude the analysis.

### Assumptions, limitations, and ethical awareness

#### Assumptions

An assumption adds uncertainty to a research study, so assumptions are articulated in advance of the data collection process and measures are taken to minimize the impact of uncertainties. Generally, participants are assumed to be able to provide reliable and valid descriptions of their experiences. Furthermore, the participant’s ability to provide an honest and detailed explanation is assumed (Leedy & Ormrod, 2014). Finally, anonymity and confidentiality are the underlying themes in the research, so the assumption is that participants provided honest and comprehensive responses.

#### Limitations

There are specific limitations to every study. As this study utilises interviews, pertinent information may not have been accurately reported in the study as a result. A few points could not be considered trustworthy based on the coverage of content analysis and coding (Levitt et al., 2017). Researchers' bias could have affected the process; therefore, it is recommended that a study be evaluated according to predetermined criteria (Elo et al., 2014). As a result of the global COVID-19 lockdown scenario in early 2021, the interviews take place over videoconferencing, which may have caused communication errors due to inefficiencies in the tool.

#### Ethical assurances

In contrast to other industries, the banking industry is heavily regulated, and any breach may result in financial institutions incurring costs of millions of dollars. As a result, bankers and regulators are not very open about sharing their perspectives or commenting on the internal processes of their organisations unless confidentiality and anonymity are explicitly confirmed. As a result, confidentiality and anonymity are communicated from the research brochure to the pre-interview discussions to the interview itself. A confidentiality agreement is signed with the university regarding the confidentiality of the thesis for a period of 5 years following its submission as a last step to protect the privacy of participants (Goyal et al., 2015).

## Results

This multi-case study is conducted to explore the adoption of digital technologies, soft information, and Big data in a bank's SME credit evaluation process and address the operational challenges associated with SME financing for a bank. This study is designed to identify the extent to which Indian banks have adopted digital technologies in the evaluation of SME credit (Rogers, 2005). The study also aims to understand the use and content of soft information while adopting digital tools and the factors that may influence the implementation of Big data (Baig et al., 2019; Yosano & Nakaoka, 2019).

**Fig. 6:**
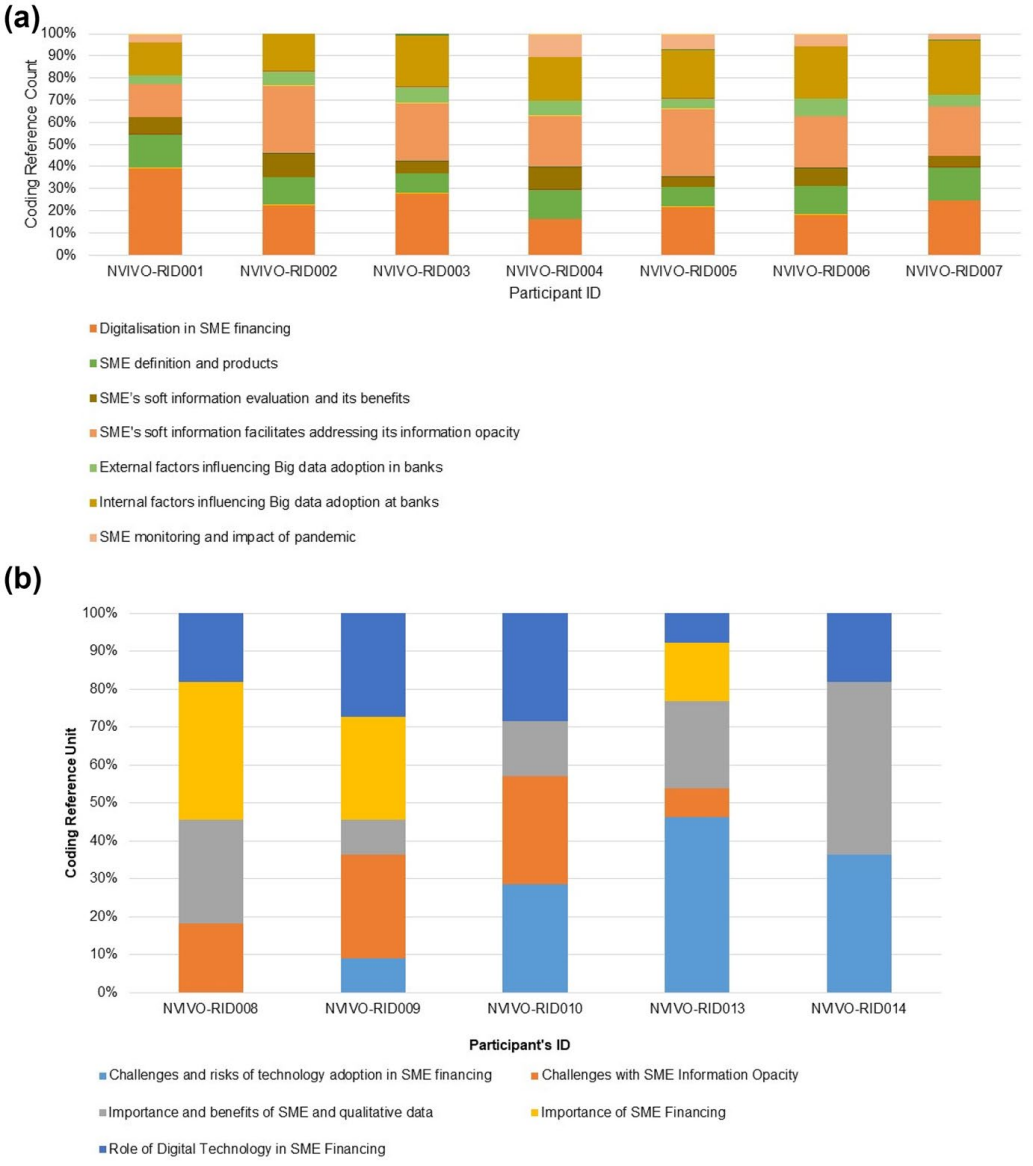
**a** A visual representation of the themes from SME financing bankers’ data and the content coverage. **b** A visual representation of the themes from regulator’s data and the content coverage. Source: By the author using NVIVO.

In this study, seven themes have emerged that are comparable with the literature and consistent with the conceptual theoretical framework of the study. These themes are (a) digitalisation improves productivity in SME financing, (b) hardening of soft information using digital tools, (c) SME’s soft information facilitates addressing its information opacity, (d) SME’s soft information evaluation and its benefits, (e) perceived internal factors influencing Big data adoption in banks, (f) perceived external factors influencing Big data adoption in banks, and (g) SME monitoring and the impact of the pandemic. The below chart represents the content coverage in terms of no. of reference by the themes (refer to Fig. 6a and b).

Research question one (RQ1) yields two main themes (refer to Table 2). Research question two (RQ2) yields two main themes (refer to Table 3). Research question three (RQ3) yields three main themes (refer to Table 4). Discussion with regulators yields five main themes (refer to Table 5).

**Table 2:** Themes for digitalisation/adoption of digital tools in SME financing

Themes	Categories	References
Digitalisation in SME financing	Information for credit evaluation	7
	Information evaluation	7
	Digital tools in SME financing	7
	Information production	6
	Decision-making	3
	Challenges with SME information opacity	2
SME definition and products	Definition of SME organisations	7
	Products offered to SME	7

Source: By the author

**Table 3:** Themes for SME’s soft information and its benefits

Themes	Categories	References
SME's soft information facilitates addressing its information opacity	Business and management leadership	7
	Network or alliance/partnership	7
	Organisational system	7
SME’s soft information Evaluation and its benefits	Benefits and influence of soft information	7
	Evaluation of soft information	4

Source: By the author

**Table 4:** Themes for internal and external factors influencing Big data adoption

Themes	Categories	References
Internal factors influencing Big data adoption at banks	Innovation factors	7
	Technology factors	7
	Organisational factor	7
External factors influencing Big data adoption in banks	Environment factor	7
SME monitoring and impact of pandemic	Impact of pandemic on SME	3
	Monitoring of SME	3

Source: By the author

**Table 5:** Themes emerged from discussions with regulatory & policy development bodies

Themes	Categories	References
Importance and benefits of SME and qualitative data	Benefits of qualitative data	5
Role of digital technology in SME financing	Digital technology role	5
Challenges with SME information opacity	SME information challenges	4
Challenges and risks of technology adoption in SME financing	Risks with Big data technology adoption in SME financing	4
	Challenge of technology in SME	2
Importance of SME financing	Importance of SME financing	3

Source: By the author

## Discussion

Challenges with SME financing are multi-fold and not linked to only one source. From the bank’s perspective, the first challenge is the operational capacity to collect soft information and transmit it within the management hierarchy for decision-making purposes. The second challenge arises from processing a large amount of SME information (Big data). Finally, the challenge arises from addressing SME information opaqueness and the most important soft information attributes.

### State of adoption of digitalisation by banks in SME credit evaluation

**Fig. 7:**
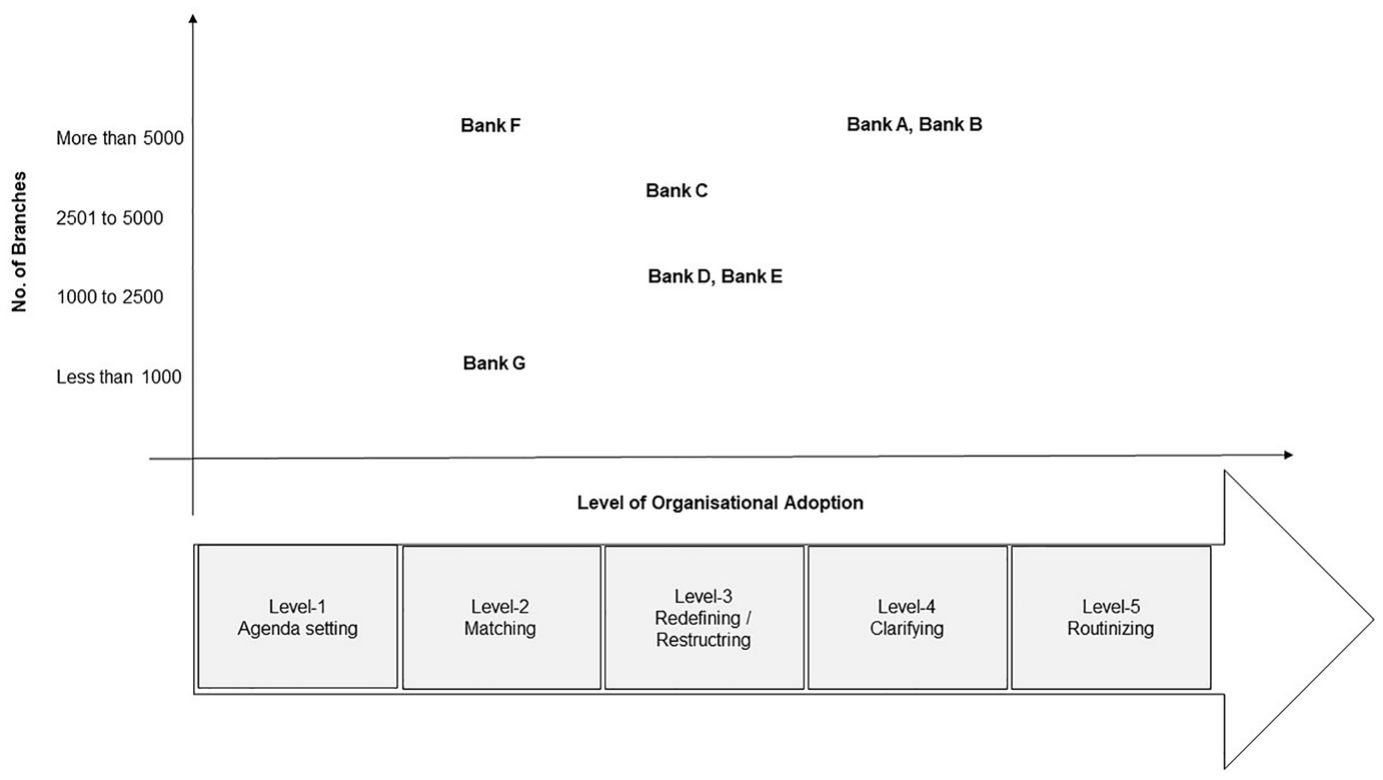
State of adoption of digitalisation in SME credit evaluation process for Indian banks. Source: By the author

This study provides a unique insight into the level of organisational adoption of digital tools and technologies by each bank in evaluating small and medium businesses. By applying Roger's (2005) parameters, the following chart is developed (refer to Fig. 7) in which the x-axis indicates the degree of adoption and the y-axis indicates the number of bank branches (size of the bank).

### Commonly perceived SME soft information attributes in SME credit evaluation

RQ2 guides data collection to find evidence of digitalisation, the content of soft information, and its benefits to address SME information opacity while understanding how bankers perceive soft information in India. According to Diamond (1984) and Berger and Black (2011), the collection of soft information is a costly process; therefore, it is more cost-effective to utilise soft information for large loans if hard information is available (Berger & Black, 2011; Diamond, 1984). Interestingly, all of the participating banks use soft information for lending to SMEs, demonstrating that the results are contrasting. The study also reveals that the percentage of banks using soft information varies between 30 and 50%, depending on their risk appetite and market focus.

**Fig. 8:**
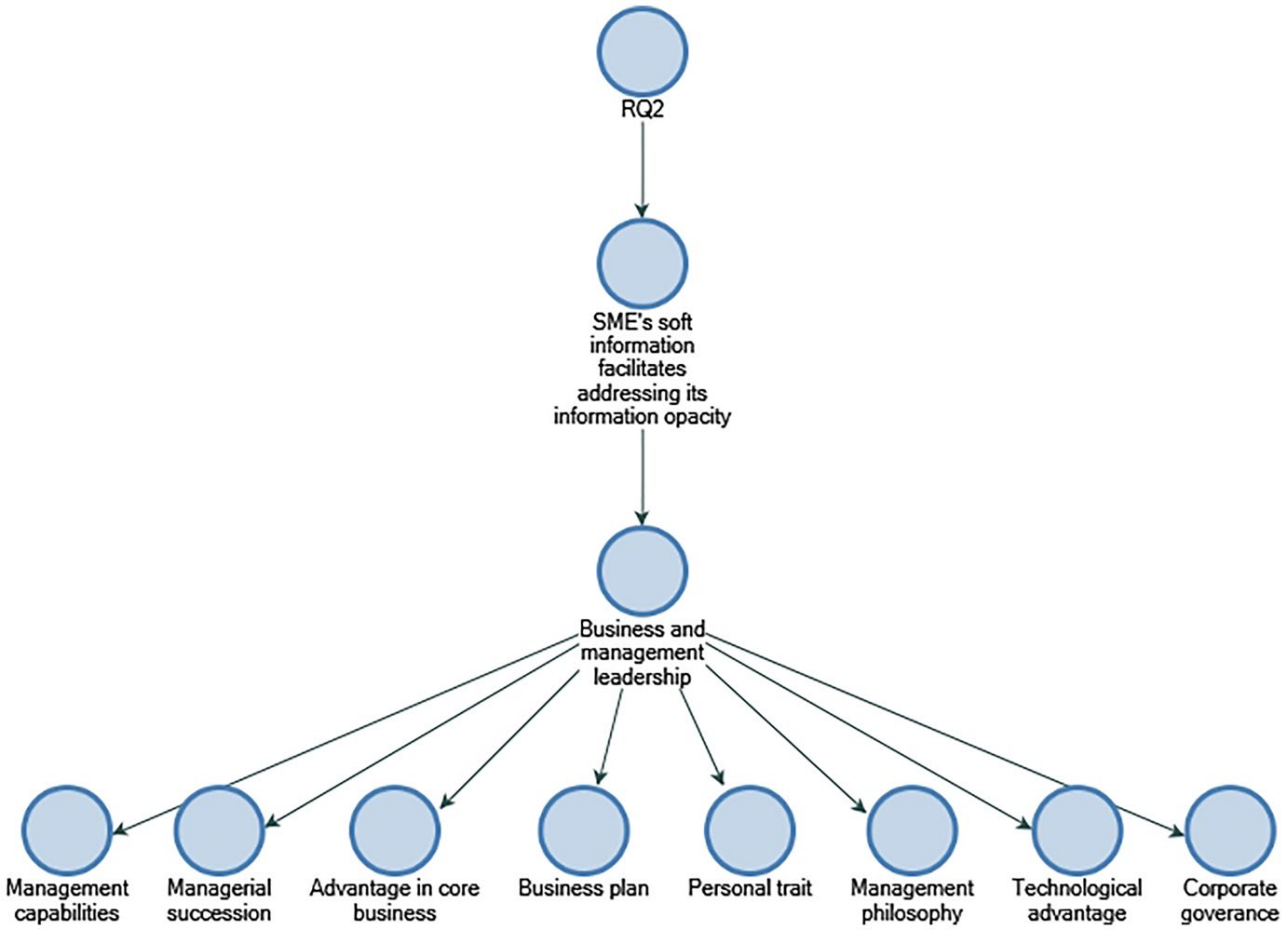
Business management and leadership soft information attributes in descending order by number of references. Source: By the author using NVIVO

**Fig. 9:**
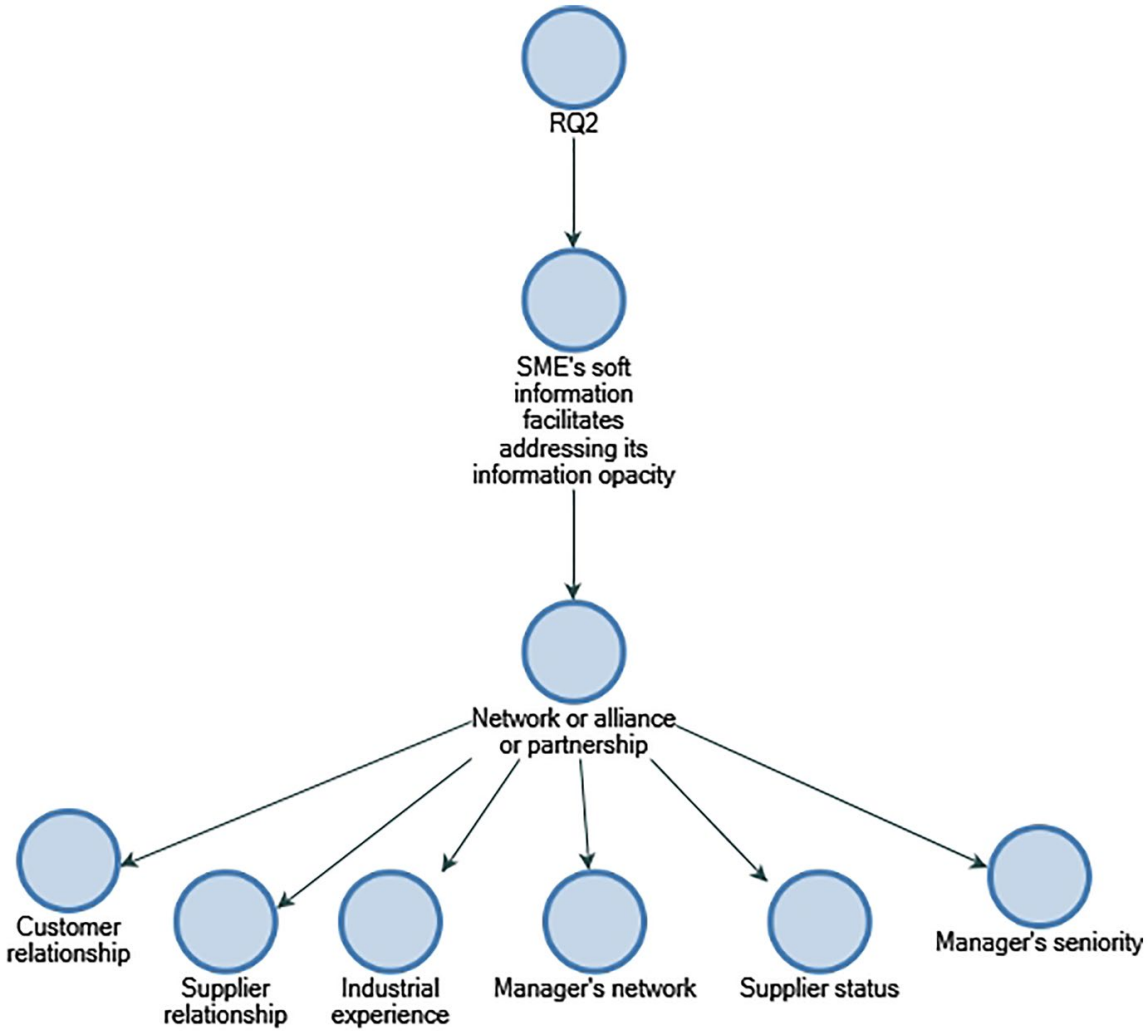
Network or alliance/partnership soft information attributes in descending order by number of references. Source: By the author using NVIVO

In line with Yosano and Nakaoka's (2019) findings, the results of RQ2 show that business and management leadership are the most significant soft information factors for Indian bankers to address the information opacity of SMEs. The results also indicate that supplier relationships, customer relationships, business plans, and managerial succession play a significant role in credit assessment for Indian small- and medium-sized businesses (refer to Figs. 8 and 9).

### Commonly perceived factors influencing Big data adoption in SME credit evaluation

**Fig. 10:**
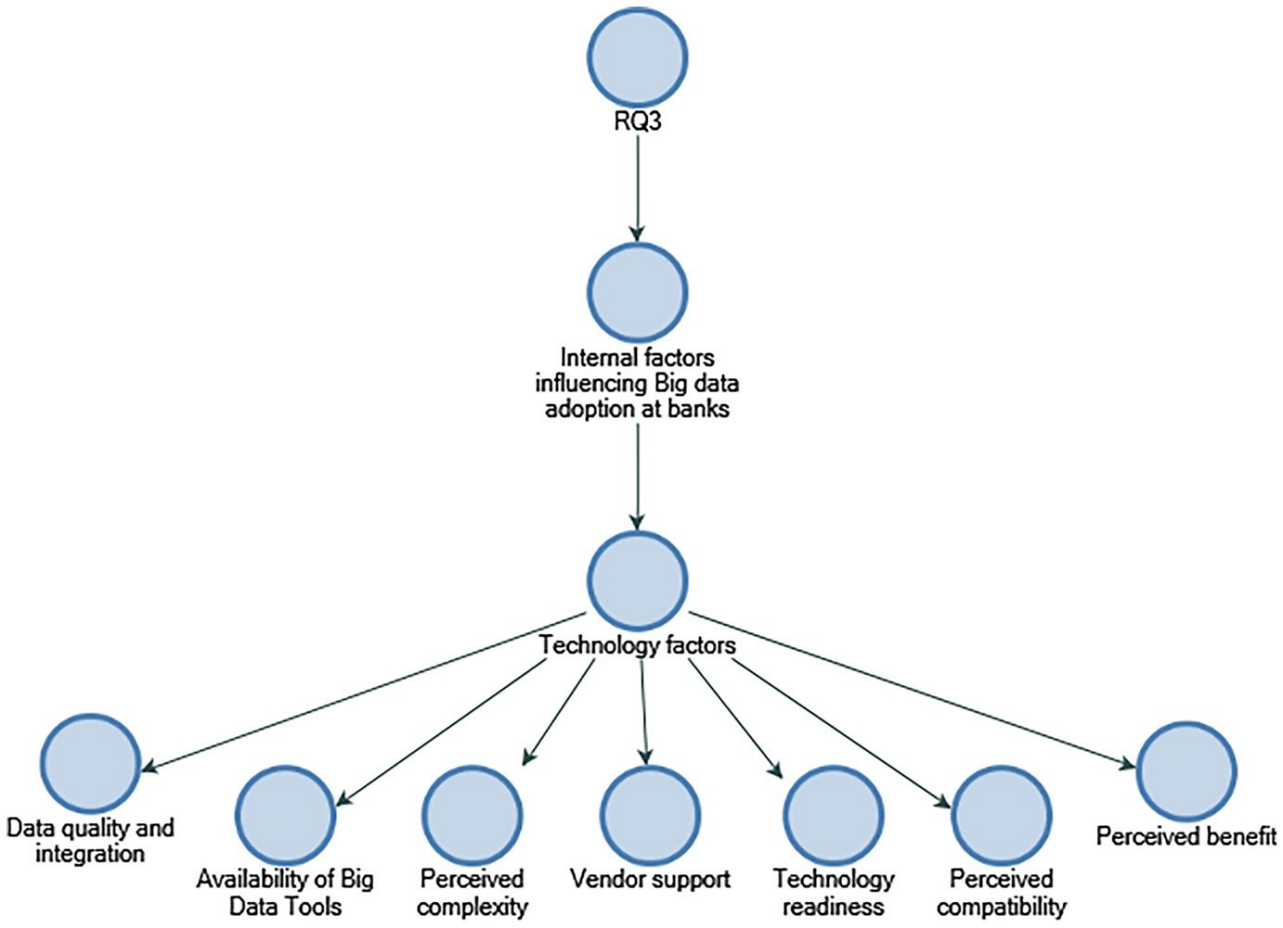
Technology factors influencing adoption of Big data in descending order by number of references. Source: By the author using NVIVO

**Fig. 11:**
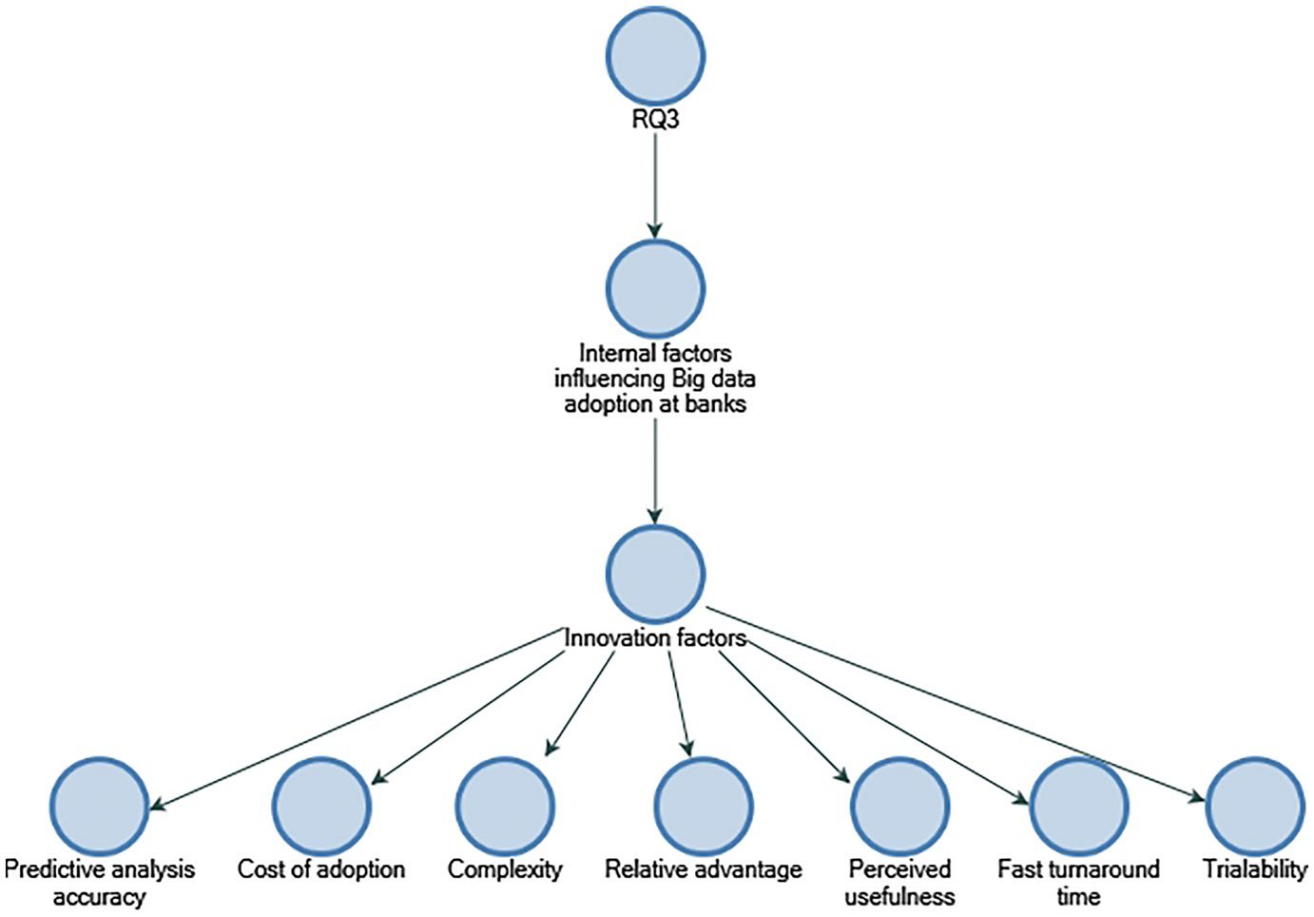
Innovation factors influencing adoption of Big data in descending order by number of references. Source: By the author using NVIVO

**Fig. 12:**
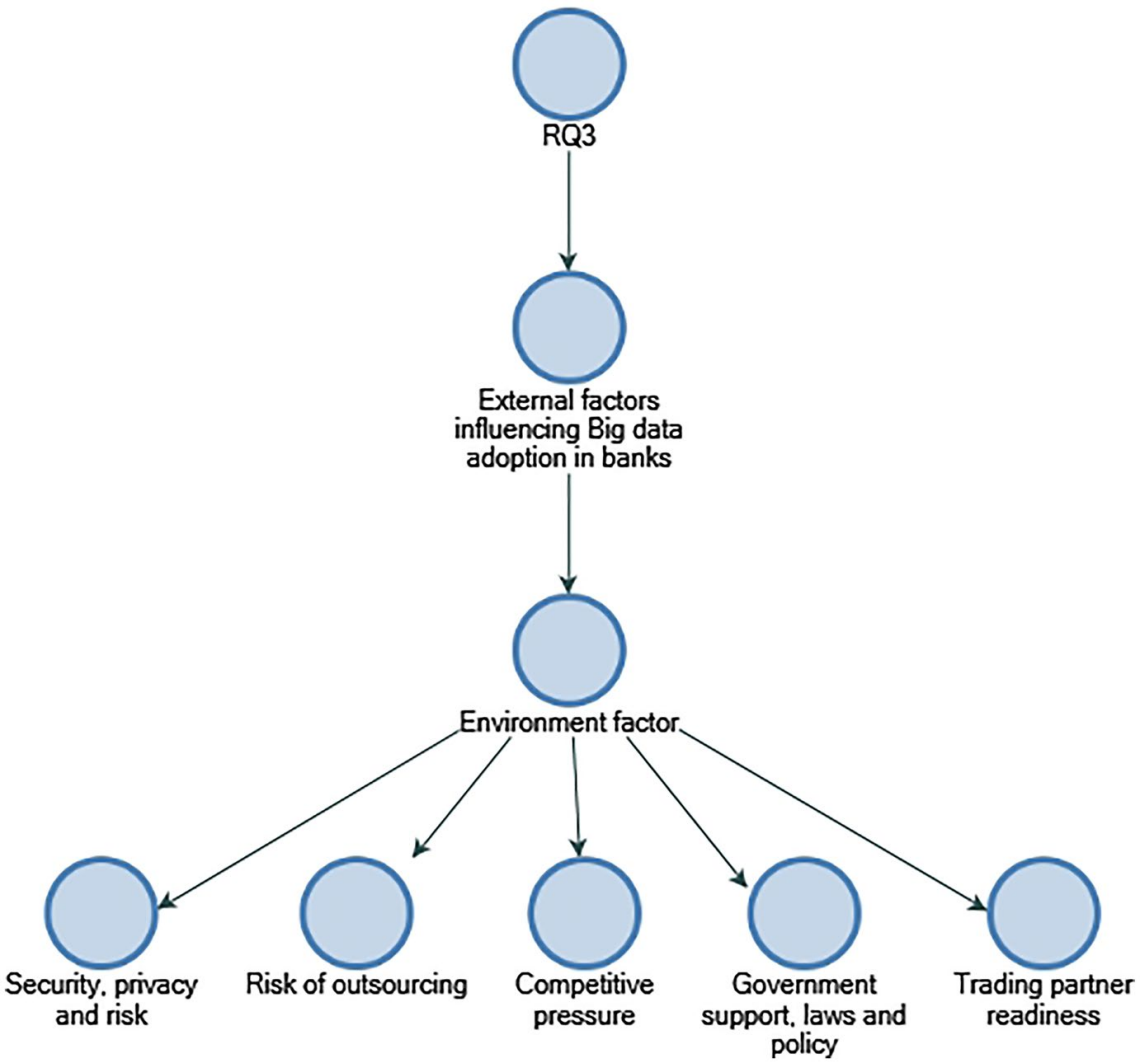
Environment factors influencing adoption of Big data in descending order by number of references. Source: By the author using NVIVO

A key objective of RQ3 is to collect data on the use of Big data in creditworthiness assessment and identify the internal and external factors that influenced its application. The participants indicate that different data and analytics systems are used by banks to assess creditworthiness, and the banks collect, verify, and store customer information from diverse sources. According to La Torre et al. (2010), banks should concentrate on small- and medium-sized businesses and align organisational resources; in addition, EBA (2018) stresses that financial institutions possess a large amount of verified, trusted, and audited data in addition to stressing the importance of utilising Big data for financial institutions. This study finds out that perceived internal factors influencing the implementation of Big data in banks included "data quality and integration", "cost of adoption", "availability of Big data tools", and "predictive analytics accuracy" (refer to Figs. 10 and 11). As the findings are not entirely consistent with those identified by Baig et al. (2009), it may be that the context of this study is a contributing factor. As far as perceived external factors are concerned, they include "risks of outsourcing" and "security, privacy, and risk". Considering external factors, the findings of "security, privacy, and risk" are consistent with Baig et al. (2019), suggesting that it could be industry-neutral and influential factors among organisations that have adopted Big data (refer to Fig. 12).

### Commonly perceived risks associated with Big data adoption in SME credit evaluation

**Fig. 13:**
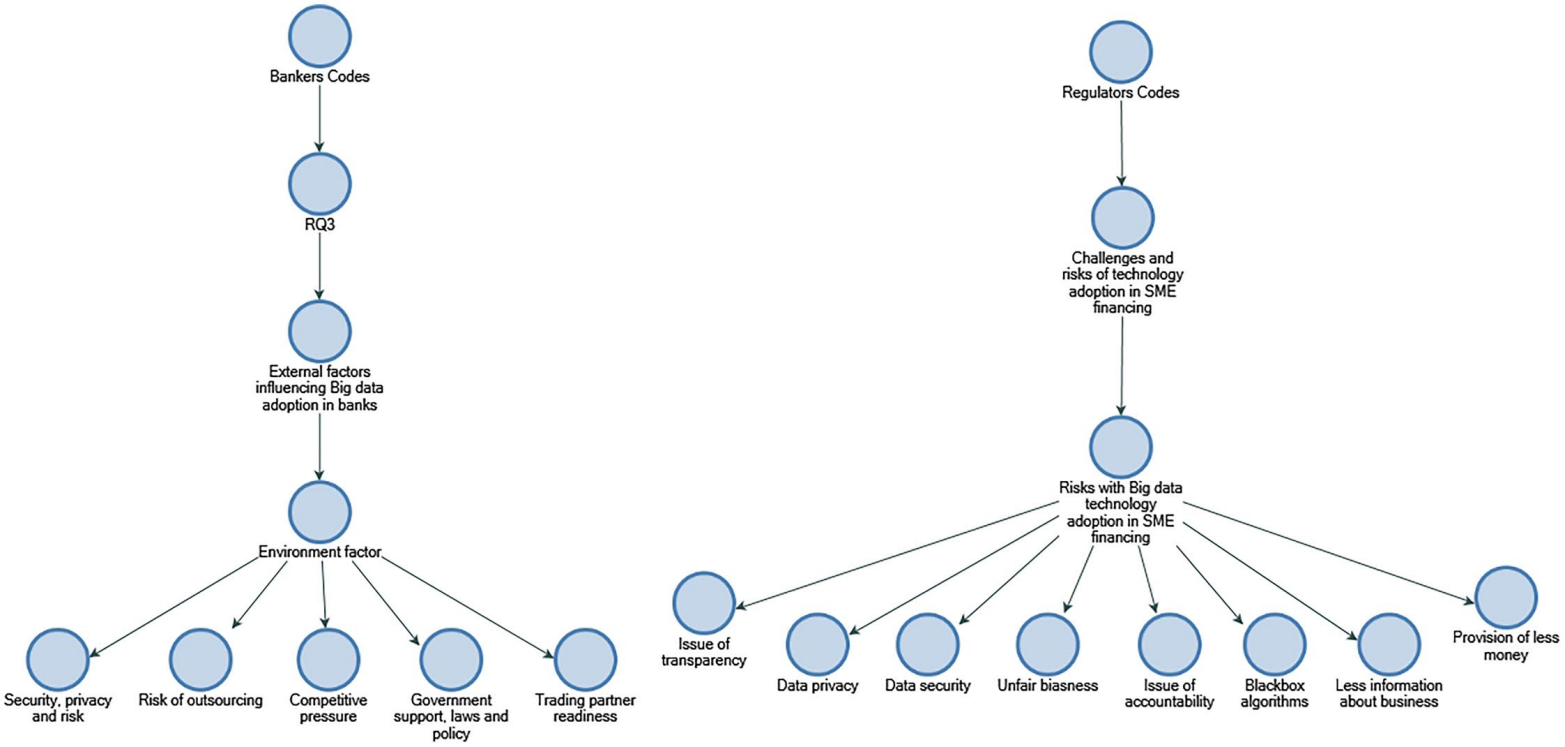
Commonly perceived risks associated with Big data adoption between bankers and regulators in descending order by number of references. Source: By the author using NVIVO

The participant of this study provides a unique insight into commonly perceived risks associated with the adoption of Big data tools in SME credit evaluation resonating between bankers and regulatory specialists. While discussing the key risks associated with the adoption of Big data tools in SME credit evaluation, bankers commonly perceive "security, privacy, and risk" and "risk of outsourcing" as the most important. On the other hand, regulatory specialist discusses the "issue of transparency", "data privacy", and "data security" as commonly perceived most important risks with banks' adoption of Big data tool for credit evaluation SMEs (refer to Fig. 13).

### Commonly perceived benefits of soft information

**Fig. 14:**
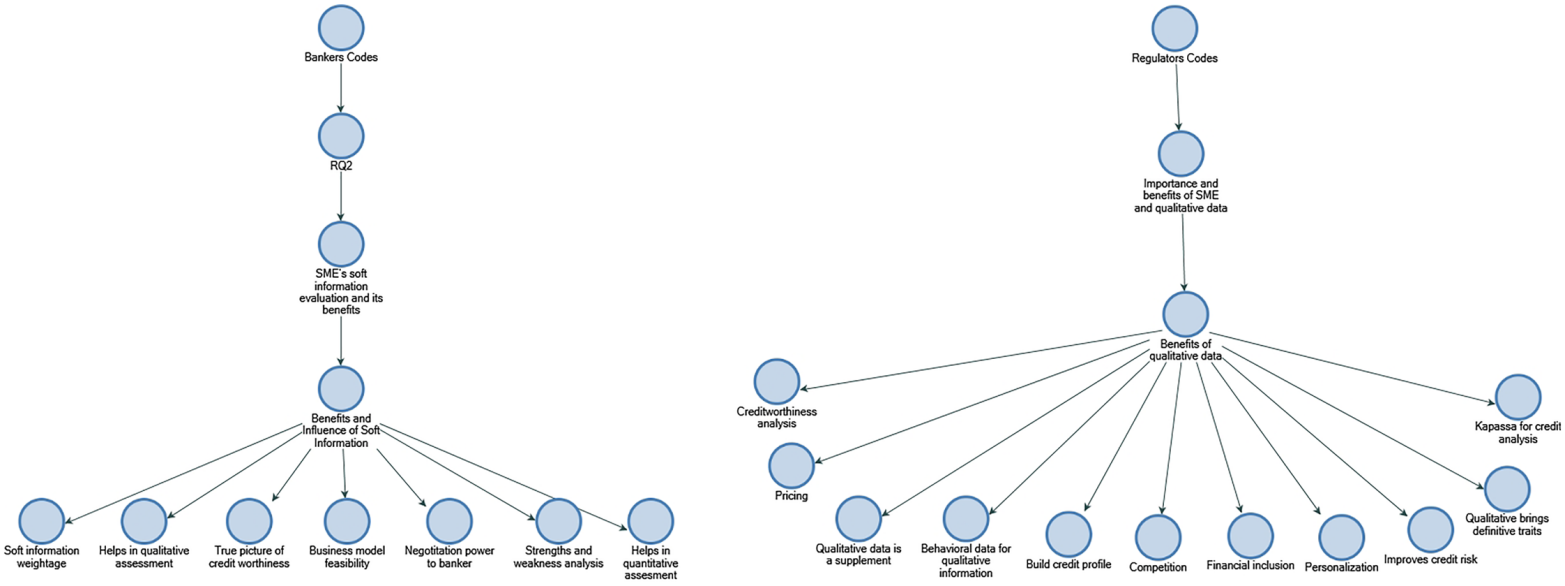
Commonly perceived benefits of soft information between bankers and regulators in descending order by number of references. Source: By the author using NVIVO

The participants of this study provide a unique insight into commonly perceived benefits of SMEs’ soft information resonating between bankers and regulatory specialists. These insights are instrumental in laying out the practical recommendation to SME lenders, banks, bank regulators, and policymakers with steps to useful action. While discussing the commonly perceived benefits of SME’s soft information in SME credit evaluation, bankers commonly perceive "soft information weightage" and "helps in qualitative assessment" as the most important. On the other hand, regulatory specialist discusses "[useful in] creditworthiness analysis", and "[loan] pricing" as commonly perceived most important benefits of soft information for SME credit evaluation (refer to Fig. 14).

## Implications

As a systemically important industry, banking and finance have been highly regulated with strict confidentiality and non-disclosure rules. As a result, researchers are unable to obtain the most current data and information from bankers or lending organisations in such a scenario. In the literature, there have been very few studies that have gathered the latest information regarding banks' evaluations of small and medium enterprises. This study provides insight into bankers' views about the adoption of digital technologies in the assessment of small- and medium-sized enterprises (SMEs) that represent a significant contribution to the theory of credit evaluation.

### Implications for SME financing research

According to academics, there are two major research purposes: first, to contribute to the development and conceptualisation of theory through empirical and conceptual research, and, second, to contribute to practice (Silverman, 2017). This study provided several practical implications for banks financing small- and medium-sized businesses based on its findings.

#### Conceptual theoretical framework

This study provides an important contribution to theoretical knowledge by proposing a conceptual theoretical framework. Researchers can use the framework to evaluate contemporary and critical areas of research, i.e., digitalisation, credit evaluation, soft information, and Big data technologies within financial institutions. In the framework, diverse yet highly related subjects have been brought together in the context of SME credit evaluation. In addition, there is limited evidence about supply side issues in the SME financing industry. Therefore, the proposed conceptual theoretical framework facilitates researchers to study the SME credit evaluation process in the financial institutions.

#### Hardening of soft information

The study results reveal limited yet significant evidence for "Hardening of soft information" during the process of producing soft information with two large banks. In accordance with other study findings, large banks are more likely to adopt information technology than smaller banks, and larger firms (irrespective of market power) are more likely to innovate in SME financing (Akhavein et al., 2005; Marinč, 2013; Schumpeter, 1983). Additionally, there are only a few studies in the literature that demonstrate lenders' ability to harden soft information to their advantage (Agarwal & Hauswald, 2010; Filomeni et al., 2016; Hattori et al., 2015).

Two areas are further examined with limited but significant evidence emerging. A participant notes that the hardened soft information could be easily transmitted within the bank and increased the accuracy of the internal rating of small- and medium-sized enterprises (Godbillon-Camus & Godlewski, 2005). One participant highlights using a traditional rating-based approach for financing SMEs that integrates soft information and addresses its constraints (Matthias et al., 2019).

#### Soft information usage, factors, and attributes

In their SME credit evaluation process, all participants confirm varying usage of soft information and acknowledge that it has a significant impact on decision-making and setting up credit conditions. In contrast to the findings of (Berger & Black, 2011; Tsuruta, 2019), this study finds that large and small banks consistently used soft information when lending to small and opaque borrowers.

It is evident from the results that two soft information factors are commonly perceived: "network or alliance/partnership" and "business and management leadership". Based on extant literature, "networks or alliances/partnerships" and "business and leadership" factors reduce lender losses (Yosano & Nakaoka, 2019). It should be noted that Matthias et al. (2019) argue that credit risk management cannot be generalised across countries, especially when soft and hard information is combined (Matthias et al., 2019). There are four commonly perceived soft information attributes within these factors: supplier relationship, customer relationship, business plan, and managerial succession. One of these attributes, business plan, is consistent with the literature on the subject as well. With regards to the "business plan" attribute, Yosano and Nakaoka (2019) argue that SMEs are informationally opaque due to a lack of track records that require lenders to obtain private and proprietary information to assess the stability and growth prospects of their businesses. Consequently, the business plan has been essential to SMEs obtaining financing from lenders as a result.

### Implications for the SME credit managers

In this study, the overall theme is centred around three essential steps in the evaluation of SME credit: the production, transmission, and processing of soft information by banks. In utilising soft information to resolve the information opacity of SMEs, banks are faced with a primary productivity constraint. Banks can benefit from implementing these recommendations in their business practices to better serve SMEs and support the socio-economic goal of the inclusion of financial services in India. A significant contribution can be made by the findings of this study to Indian banks' credit managers responsible for securing credit for SMEs.

The immediate priority recommendation for SME credit managers is that the soft information attributes for SME credit managers must be reprioritised, starting with "network or alliance/partnership" and "business leadership". The "network or alliance/partnership" of SMEs can be characterized as publicly accessible soft information. SMEs conduct transactions with their customers/suppliers using online B2B trade platforms, which are a new and emerging source of publicly accessible soft information. It is possible for SME credit managers to collaborate with such platforms to gain access to SMEs' soft information and private hard information.

The short-term priority recommendation for SME credit managers would be that as custodians of SME credit evaluation, credit managers play a crucial role. Using digital tools, SME credit managers can propose and lead soft information hardening in the SME credit evaluation process. Adopting new tools and technologies requires business process redesign. The hardening of soft information enables it to be transmitted within the organisation hierarchy of the bank for decision-making purposes as well as easier to store. As such, the second recommendation for SME credit managers is to propose or lead the implementation of developing an internal credit rating for SMEs based on soft data or a rating-based methodology. By implementing these two measures, the bank would be able to improve its coverage of the SME market and identify potential SMEs by applying a risk-based approach.

The medium-term priority recommendation for SME credit managers would be that it is becoming increasingly common for banks to manage massive customer data to derive value from the multitude of data sources and types of information that are available. In the medium term, SME credit managers can influence the higher management to switch towards Big data implementation that facilitates enhancing the value that the bank derives from customer relationships. The margins from SME financing will consistently diminish as competition intensifies in the market. For banks to make the SME customer relationship profitable, it may also be necessary to cross-sell products and services. For a bank to move from data-driven decision-making to data-driven customer relationship management, SME credit managers can demonstrate the cost–benefit of implementing Big data to higher management and executives.

The recommendation for the regulatory and policy development bodies would be to encourage the use of soft information. Credit rating agencies have been a key contributor to the widespread of credit to corporations and individual consumers for the last 2–3 decades (Carruthers & Cohen, 2010; Kiviat, 2017; O'Neil, 2017). In India, credit rating agencies can devise strategies to include public soft information in public credit scores, such as CIBIL, which eliminates scepticism and encourages wider usage of soft information.

### Limitations

This research contributes to the emerging field of digitalisation in SME financing by addressing some of the current research gaps, namely soft information constraints, soft information content, and the adoption of digital technologies in SME financing. As with any research, this study has its limitations as well.

First, the primary method of data collection in this study is semi-structured interviews, which are subject to bias on the part of participants and researchers. The participant’s bias has been addressed by making sure participants know that their data are truly confidential which enables them to reveal the truth, even if they do not consider it to be a great social advantage. I have made a reasonable effort with the university to ensure the confidentiality and anonymity of the study. In addition, the researcher’s bias has been minimized by providing a clear and consistent experimental setting for each participant.

Second, India has 35 banks covering both public and private sectors, but this study only examines seven of them, which do not represent the market as a whole. To address that the sample includes top Indian banks, which represent 65%–70% of the market capitalisation of the Indian banking industry. Moreover, theoretical sampling variables, branch count, and type of bank (public or private) are used to develop generalizable comparisons among cases.

Third, banks are structured organisations with well-defined hierarchies. For credit assessment, the management hierarchy includes loan officers, supervisors, managers, and senior managers. The study, however, concentrates exclusively on middle management bankers, who are responsible for financing SMEs. One might argue that there is no evidence of lenders' perspectives on SME financing in the literature. The study is one of the few that has attempted to capture the views of the bankers, who are also the decision-makers.

Fourth, based on the soft information content, two perceived factors and four perceived attributes represent a limited view of middle managers from a few specific banks in India. However, one might argue that it is legitimate to rely on a qualitative exploratory approach due to the lack of empirical studies in extant “soft information in SME financing” research as well as the limited number of comparable studies (only three are found) on this particular topic. In order for the results introduced in this study to be generalizable in the Indian context, future quantitative research may be required.

Finally, when it comes to attributes that influence Big data adoption, the five perceived attributes represent a limited view of middle management bankers in a few specific Indian banks. It has been addressed by utilising data triangulation, which incorporates the views and perceptions of regulatory bodies closely monitoring Big data adoption.

### Implications for future research

Future studies could also explore the boundary conditions, facilitating the participation of more banks, including regional banks that may have a significant role in SME financing. These banks may offer insight into the adoption of digital technologies. Research that collects lender perspectives on SME financing can validate findings on soft information hardening, soft information factors and attributes in SMEs, and factors influencing the adoption of Big data. Consequently, it is recommended that the stated problem be explored using mixed methods or quantitative methods. Since this study is conducted using a qualitative approach, the only source of data is interviews, which limited the ability to evaluate the findings from a statistical perspective.

Unlike other industries, the evidence of the lender’s view is limited due to the regulation of the banking and finance industry. It is therefore imperative that researchers inquire about lenders’ perceptions of lending to small and medium business enterprises. This will enable them to gain unique insights into how digital disruption is being experienced locally. Further research should examine how banks or lenders are moving towards personalisation and customer-centeredness in the SME market and how innovation in SME product development is occurring. It is pertinent to consider comparing the results and findings of similar studies conducted in other developing countries to contribute to the literature.

I sincerely hope that this research and the proposed conceptual theoretical framework will provide inspiration for future research on regional SMEs' soft information attributes and factors.

## Data Availability

Considering the financial nature of the organizations involved, the participants of this study agreed to share their personal views under the condition of confidentiality and anonymity. The data are not available publicly due to restrictions relating to the content of the data, which may compromise the privacy of research participants. Nevertheless, masked data from the study can be obtained upon request from the corresponding author, Nimbark Hardik.
